# Simulation and Experimental Verification of Magnetic Field Diffusion at the Launch Load during Electromagnetic Launch

**DOI:** 10.3390/s23188007

**Published:** 2023-09-21

**Authors:** Yuxin Yang, Qiang Yin, Changsheng Li, Haojie Li, He Zhang

**Affiliations:** 1 Ministerial Key Laboratory of ZNDY, Nanjing University of Science and Technology, Nanjing 210094, China; 317101010026@njust.edu.cn (Y.Y.); haojieli@njust.edu.cn (H.L.); hezhangz@njust.edu.cn (H.Z.); 2 Southwest Institute of Technical Physics, Chengdu 610041, China; xtsxwbyinqiang@163.com

**Keywords:** electromagnetic rail launch, magnetic diffusion, velocity skin effect, coupled simulation, projectile-borne measurement

## Abstract

The unique magnetic field environment during electromagnetic launch imposes higher requirements on the design and protection of the internal electronic system within the launch load. This low-frequency, Tesla-level extreme magnetic field environment is fundamentally distinct from the Earth’s geomagnetic field. The excessive change rate of magnetic flux can readily induce voltage within the circuit, thus disrupting the normal operation of intelligent microchips. Existing simulation methods primarily focus on the physical environments of rails and armatures, making it challenging to precisely compute the magnetic field environment at the load’s location. In this paper, we propose a computational rail model based on the magneto–mechanical coupling model of a railgun. This model accounts for the dynamic current distribution during the launch process and simulates the magnetic flux density distribution at the load location. To validate the model’s accuracy, three-axis magnetic sensors were placed in front of the armature, and the dynamic magnetic field distribution during the launch process was obtained using the projectile-borne-storage testing method. The results indicate that compared to the previous literature methods, the approach proposed in this paper achieves higher accuracy and is closer to experimental results, providing valuable support for the design and optimization of the launch load.

## 1. Introduction

Electromagnetic rail launch technology represents a specialized launching technique that utilizes electromagnetic force as its energy source. In comparison to conventional propulsion methods, electromagnetic railguns offer advantages such as high muzzle kinetic energy and controllable speed [1,2,3,4]. However, the electromagnetic launch process generates an extremely harsh environment within the bore, characterized by pulsed high magnetic fields in the Tesla range [5]. The magnetic field in front of the armature can reach a peak of 10 T, with a magnetic flux density change rate of up to 19 T/ms [6]. The magnetic field far exceeds the geomagnetic conditions and constitutes a unique extreme environment [7]. This challenging environment poses a significant threat to the safety and reliability of the electronic systems within the launch load [8]. Therefore, understanding the magnetic field environment around the load during the launch process is of paramount importance for the reliable design and protection of electronic systems. The launch load is assembled at the front of the armature and is connected via insulating supports, and its internal magnetic field environment is influenced by the electromagnetic diffusion fields generated by the rail and the armature [9]. Additionally, the high-speed relative motion between the armature and the rail induces the velocity skin effect, thereby impacting current distribution [10]. Existing commercial simulation software struggles to address high-speed sliding electrical contact issues and is prone to grid distortions during the simulation process, thus increasing research complexity and costs [11].

Currently, scholars primarily focus on the electromagnetic field distribution between the armature and the rail [12,13,14,15,16], and there is relatively limited research on the magnetic field environment of the launch load. Lin et al. established an electromagnetic field simulation model based on a hybrid finite element and boundary element method [17] but did not consider the transmission relationship of the magnetic field from the armature to the load. Li et al. calculated the magnetic flux density distribution along the projectile axis position using an analytical method [18], but the input current and rail–armature model were simplified. Yin et al. developed an equivalent model for the armature and rail under motion conditions [19]. However, their approach employed an average rail calculation method and did not thoroughly investigate the influence of the velocity skin effect on current distribution. The authors conducted magnetic field simulations for the load position previously. The model was static and unable to accurately simulate the dynamic changes in the magnetic field. In terms of experiments, Schneider et al. have developed giant magnetoresistance sensors capable of measuring transient strong magnetic fields [20]. They measured the magnetic field near the rails and at the rear of the armature, but they did not focus on the magnetic field distribution around the load position. Hundertmark et al. tested the magnetic field distribution of the projectile’s position during the launch process using wireless transmission [21], but the testing speed was low, and the velocity skin effect was not clearly observed. Overall, the existing literature is insufficient in studying the magnetic field environment at the load, and there are some technical limitations, which are primarily reflected in the inaccurate magnetic diffusion model of projectile position and the lack of experimental verification, among others.

In this paper, a computational method is proposed that can adaptively adjust the shape and contact length of the current distribution in the rail region during launch, aiming to minimize the computational error caused by average current density instead of actual density. Firstly, a magneto–mechanical coupling model of the rail–armature during electromagnetic launch is established. Taking into account the influence of the velocity skin effect and the dynamic changes in the contact area between the rail and armature, a rail model that adapts to the distribution of the current streamline is established, and the rail height and rail–armature contact length at each moment are treated as varying parameters for iterative calculations to obtain the magnetic field distribution at the load position. Finally, a three-axis magnetic field projectile storage system is designed, and dynamic launch experiments are conducted to obtain magnetic field data for comparison and analysis with the simulation results.

## 2. Force–Magnetic Coupling Simulation Method

**Figure 1:**
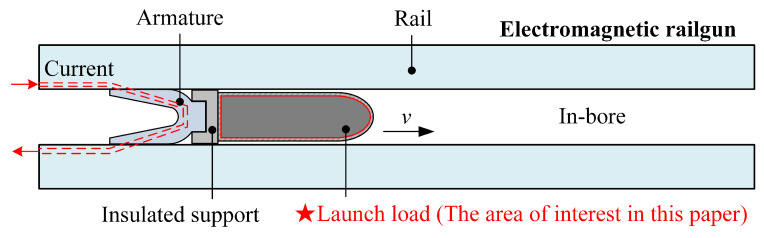
Schematic diagram of in-bore electromagnetic railgun during launch.

During electromagnetic launch, pulsed currents flow through both the rail and the armature, as shown in Figure 1. The interaction between the current and the magnetic field generates electromagnetic force, propelling the armature and the projectile in the direction indicated. Concurrently, a high-intensity magnetic field pulse is generated within the bore.

### 2.1. Electromagnetic Field Calculation Model

The pulsed current is influenced by the skin effect and the proximity effect during launch, which are primarily distributed along the inner surface and edges of the rail. Therefore, the electromagnetic field distribution inside the bore can be simplified as the process of inward diffusion along the inner surface of the rail [22]. Furthermore, due to the wavelength of the input current being much larger than the dimensions of the rail, the influence of the displacement current can be neglected, and the system is assumed to be in a quasi-static state [23]. Based on the differential form of Maxwell’s equations and Ohm’s law, it can be obtained that

(1) $$\nabla^{2}H=\sigma \mu_{0}\frac{\partial H}{\partial t}-\sigma \mu_{0}\nabla \times \left(v\times H\right)$$

where ∇ is the Hamiton counters, ***H*** is the magnetic field intensity, σ is the conductivity of the conductor, $\mu_{0}$ is the permeability of vacuum, and v is the velocity of the conductor.

In three-dimensional calculations, assuming that there are no ferromagnetic conductors in the armature and the rail (A&R), we have B instead of H. The magnetic diffusion of the rail can be expressed as [24]

(2) $$\frac{\partial B}{\partial t}-\frac{1}{\sigma_{r}\mu_{0}}\left(\frac{\partial^{2}B}{\partial x^{2}}+\frac{\partial^{2}B}{\partial y^{2}}+\frac{\partial^{2}B}{\partial z^{2}}\right)+v_{x}\cdot \frac{\partial B}{\partial x}=0$$

where ***B*** is the magnetic flux density, $v_{x}\cdot \frac{\partial B}{\partial x}$ is the diffusion term of the velocity, and $\sigma_{r}$ is the conductivity of the rail.

Assuming that the armature is stationary and the rail has a velocity in the negative *x*-direction relative to the armature, the magnetic diffusion in the armature region can be expressed as follows:

(3) $$\frac{\partial B}{\partial t}-\frac{1}{\sigma_{a}\mu_{0}}\left(\frac{\partial^{2}B}{\partial x^{2}}+\frac{\partial^{2}B}{\partial y^{2}}+\frac{\partial^{2}B}{\partial z^{2}}\right)=0$$

where $\sigma_{a}$ is the conductivity of the armature.

The above equation can be solved by setting the boundary conditions and the initial value of B. The current density J in the *x*-, *y*-, and *z*-axes can be obtained [25]

(4) $$\left\{\begin{array}{c} j_{x}=\frac{1}{\mu_{0}}\left(\frac{\partial B_{z}}{\partial y}-\frac{\partial B_{y}}{\partial z}\right)\\ j_{y}=\frac{1}{\mu_{0}}\left(\frac{\partial B_{x}}{\partial z}-\frac{\partial B_{z}}{\partial x}\right)\\ j_{z}=\frac{1}{\mu_{0}}\left(\frac{\partial B_{y}}{\partial x}-\frac{\partial B_{x}}{\partial y}\right) \end{array}\right.$$

Ignoring the magnetic flux density of the A&R in the *x*- and *y*-axes, we have

(5) $$\left\{\begin{array}{c} j_{x}=\frac{1}{\mu_{0}}\frac{\partial B_{z}}{\partial y}\\ j_{y}=\frac{1}{\mu_{0}}\frac{\partial B_{z}}{\partial x}\\ j_{z}=0 \end{array}\right.$$

### 2.2. Equivalent Circuit Model

To obtain a relatively stable acceleration environment, multiple pulse-forming units (PFUs) are generally connected in parallel to form a pulse-forming network (PFN) [26]. By controlling the discharge time of each PFU, the amplitude and pulse width of the appropriate current can be obtained. For a PFN composed of *n* capacitor module groups, the total output current of the entire pulsed power supply is the sum of the output currents of the *n* capacitor module groups. The equation for the output current can be expressed as:

(6) $$I=\left\{\begin{array}{cc} I_{1} & 0\leq t\leq t_{o1}\\ I_{1}+I_{2} & t_{o1}\leq t\leq t_{o2}\\ \cdot \cdot \cdot & \cdot \cdot \cdot \\ I_{1}+I_{2}+\cdot \cdot \cdot +I_{n} & t_{on-1}\leq t\leq t_{on} \end{array}\right.$$

where $I_{1}$, $I_{2}$, $I_{n}$ is the number of the current in each PFU, and $t_{o1}$, $t_{o2}$, $t_{on}$ is the trigger time.

**Figure 2:**
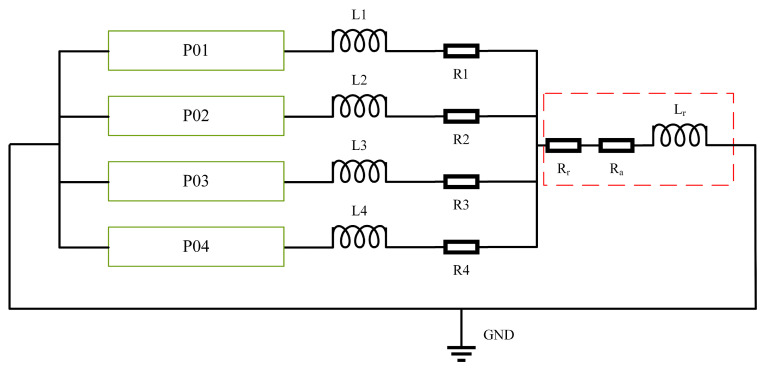
Equivalent circuit of pulse-forming network.

The equivalent circuit of the electromagnetic railgun PFN is shown in Figure 2. R1∼R4 represents the equivalent resistance of each PFU, and the resistance value is 7 mΩ. L1∼L4 represents the equivalent inductance, and the value is 0.6 μH. $R_{r}$ and $L_{r}$ are the resistance and inductance of the rail, respectively; $R_{a}$ is the resistance of the armature. The trigger time of each PFU is shown in Table 1.

### 2.3. Magneto–Mechanical Coupling Calculation Model

**Figure 3:**
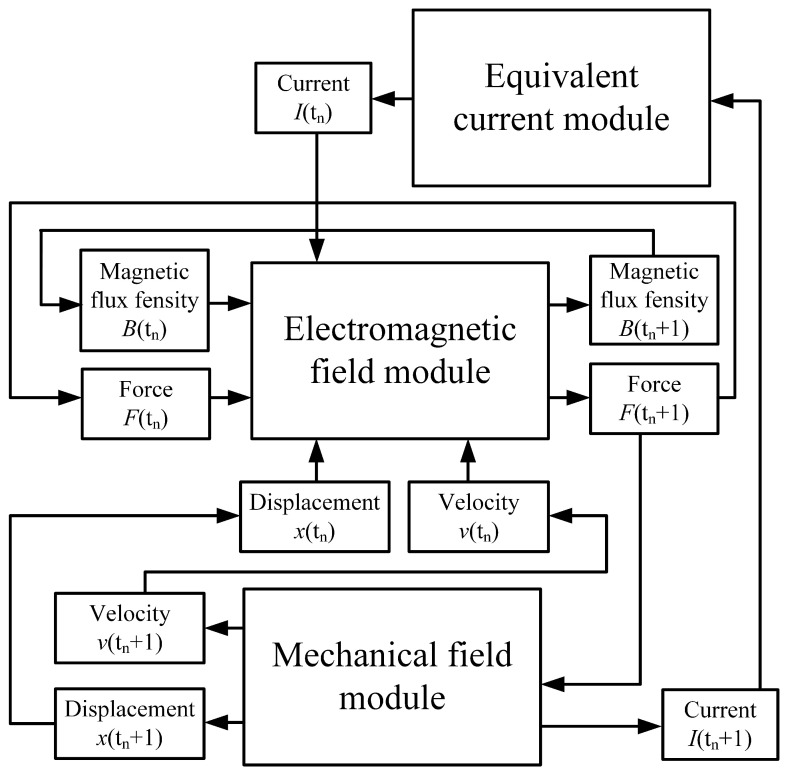
Magneto–mechanical coupling calculation model.

Based on the above model, a magneto–mechanical coupling calculation model can be established by iteratively calculating and updating the results through a looping process. The time of the force–magnetic coupled simulation is discretized as $t_{1}$, $t_{2}$, *…*, $t_{n}$, $t_{n+1}$, *…*, $t_{N}$. The coupling relationship between the electromagnetic field, force field, and equivalent circuit module is shown in Figure 3. The current density, magnetic flux density, velocity, displacement, and current are used as bridges to construct the coupling relationship between the three modules.

The Lorentz force on the armature is obtained by integrating the product of the magnetic flux density and current density at each point inside the armature. It is mainly divided into the axial Lorentz force $f_{x}$ of the armature and the radial Lorentz force $f_{y}$ of the rail as follows

(7) $$f_{x}=-\int_{V_{a}}B_{z}j_{y}dV$$

(8) $$f_{y}=\int_{V_{a}}B_{z}j_{x}dV$$

where the *z*-axis magnetic induction $B_{z}$ of the A&R is obtained by solving the magnetic diffusion partial differential equations in Equations (5) and (6). The variables $j_{x}$ and $j_{y}$ are the current density distributions of the A&R, respectively, which are solved according to Equation (8). $V_{a}$ is the armature volume.

According to the equivalent relationship between electromagnetic force and Lorentz force in the armature dynamics model, Equation (8) is substituted into the mechanical preload $F_{\mathrm{NM}}$ to obtain [27]

(9) $$F_{f}=\mu_{s}F_{N}=\mu_{s}\left(F_{\mathrm{NM}}+f_{y}\right)$$

where $\mu_{s}$ is the static friction coefficient, and $F_{N}$ represents the contact pressure between the A&R.

The acceleration of the armature is

(10) $$a=\frac{f_{x}-2F_{f}-F_{a}}{m}=\frac{f_{x}-2\mu_{s}\left(F_{\mathrm{NM}}+f_{y}\right)-1.1\rho_{a}S_{A}v^{2}}{m}$$

where $F_{a}$ represents the air resistance force, and $\rho_{a}$ is the air density, which is 1.29 $\mathrm{kg}/m^{3}$ under standard conditions [23]; $S_{A}$ represents the cross-sectional top surface of the armature; *v* denotes the velocity; and *m* is the armature mass.

The velocity is obtained by integrating the acceleration

(11) $$v\left(t\right)=\int_{0}^{t}adt+v_{0}$$

Also, the displacement of the armature is

(12) $$x\left(t\right)=\int_{0}^{t}v(t)dt+x_{0}$$

where $x_{0}$ is the initial displacement.

### 2.4. Armature Velocity and Current Distribution

**Figure 4:**
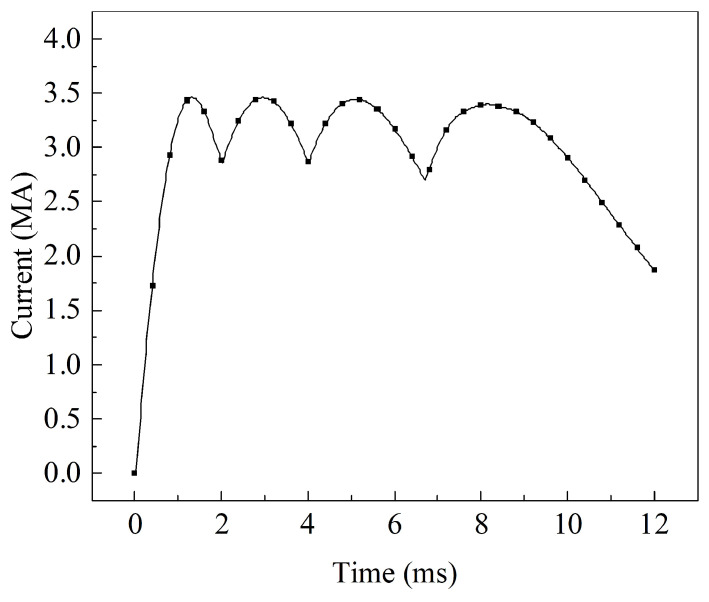
Current.

**Figure 5:**
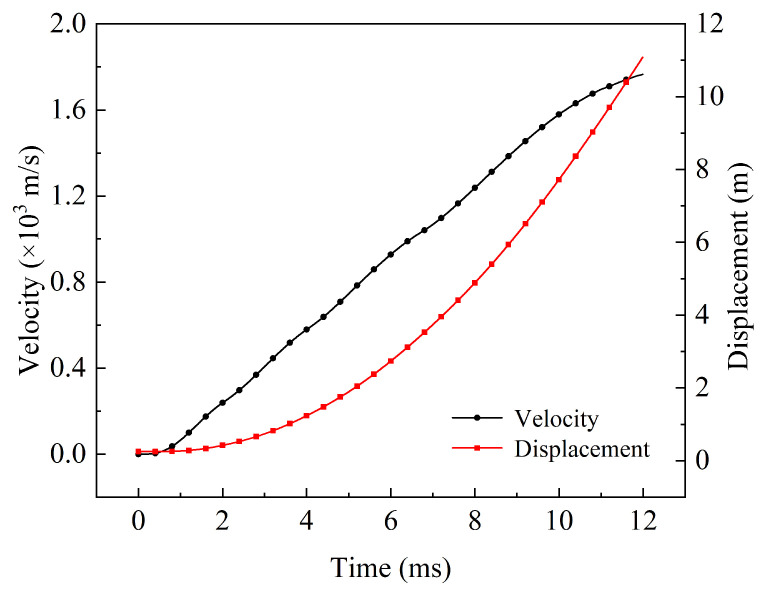
Velocity and displacement.

The current of the armature and rails can be obtained by a magneto–mechanical coupling calculation model, as shown in Figure 4. The peak current reached 3.46 MA with a duration of 12 ms. The first to fourth pulse peaks were 3.46 MA, 3.45 MA, 3.44 MA, and 3.39 MA, with peak times of 1.2 ms, 2.9 ms, 5.0 ms, and 8.1 ms, respectively. The first to third pulse valleys were 2.86 MA, 2.85 MA, and 2.70 MA, with valley times of 2 ms, 4 ms, and 6.7 ms, respectively. The final stage was the current decay phase, where the current dropped to 1.7 MA at 12 ms. Meanwhile, according to Equations (11) and (12), the velocity and displacement of the armature can be calculated as shown in Figure 5, with an acceleration process from 0 to 1760 m/s completed within 12 ms.

**Figure 6:**
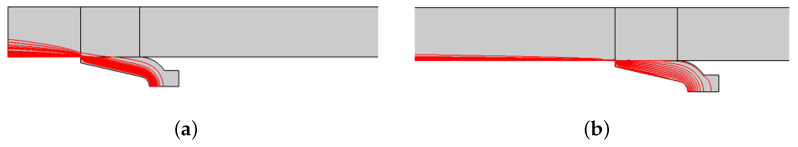
Comparison of current line distribution between two moments during launch: (**a**) 1 ms and (**b**) 8.1 ms.

In order to investigate the impact of the motion velocity on the current distribution, the current distribution of the A&R in the xy-plane under different velocities is determined using COMSOL PDE module, as shown in Figure 6. The current is 3.26 MA and the velocity is 65 m/s at 1 ms. As the velocity is low, the pulse current is mainly distributed within the inner surface of the A&R, as shown in Figure 6a. However, the current is 3.28 MA and the velocity is 1420 m/s at 8.1 ms. As the velocity increased, the current line distribution in the armature covered the entire region, as shown in Figure 6b. Meanwhile, the current in the rail became more concentrated on the inner surface, and the transmission area of the rail and armature was concentrated in a small area behind the armature tail. These simulation results are consistent with the physical interpretation of the velocity skin effect associated with increasing velocity.

## 3. Calculation Method of Magnetic Field Distribution at Launch Load

Assuming the shell material of the load is non-metallic, according to the Biot–Savart law, the magnetic flux density at any points on the rail and armature due to any current element is

(13) $$B\left(r\right)=\frac{\mu_{0}}{4\pi }\int \frac{J\left(r^{\prime }\right)\times \left(r-r^{\prime }\right)}{{\left|r-r^{\prime }\right|}^{3}}dV\left(r^{\prime }\right)$$

where *V* represents the volume of the current distribution region, $r^{\prime }$ is the vector radius from the source point (current element *J*d*V*), and *r* is the vector radius from the field point. Therefore, the magnetic flux density at the load position can be calculated based on the spatial position of the load relative to the source point and the current density components at the source point.

**Figure 7:**
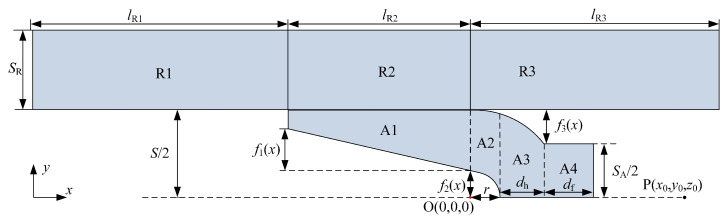
Sub-regional calculation model of the A&R.

Considering the diffusion effects of current in the A&R and the structure of a rectangular rail and C-shaped armature, the 1/2 calculation model of the A&R is established, as shown in Figure 7. To differentiate between different computing regions, the rail is divided into three regions corresponding to R1, R2, and R3. The armature is divided into four regions for calculation, where $y=f_{1}\left(x\right)$, $y=f_{2}\left(x\right)$, $y=f_{3}\left(x\right)$ represent the equations of the profile curves of the inner surface of the tail wing, the curvature circle, and the outer edge of the throat, respectively. $S_{R}$ represents the width of the rail, $l_{R1}$, $l_{R2}$, and $l_{R3}$ represent the length of the rail in R1, R2, and R3, respectively.

By utilizing the calculated current density $J_{vx}$ and $J_{vy}$ within each region, the magnetic flux density at the point $P(x_{0},y_{0},z_{0})$ is integrated, and then the vector sum is performed to obtain the magnetic flux density generated by the entire armature at point *P*. However, calculating the magnetic field at different location points requires the integration of the current density distribution within the A&R, resulting in a certain level of computational complexity. In contrast, numerical simulation methods can simplify the analytical calculation process and provide a more intuitive way to solve the spatial magnetic field distribution of the launch load. Considering that the current density of each point in the model cannot be assigned in the finite element calculation process, an equivalent method such as the average current density must be used. In this section, based on commercial COMSOL PDE module and the distribution characteristics of the current within the rails and the armature during launch, we establish a computational model that allows for the adaptive adjustment of the shape and contact length of the current density distribution area within the rails, thus minimizing the calculation error caused by using the average current density to replace the actual current density.

### 3.1. Average Current Density Method

Assuming zero current density in the *z*-direction and zero magnetic flux density in the *x*-direction of the armature as stated in Equation (5) and taking into account the symmetry of the armature about the *x*-axis, the magnetic flux density in the *y*-direction of the armature is also zero. Therefore, when calculating the magnetic flux density within the bore, only the *x*- and *y*-directional currents in the armature need to be considered to generate a *z*-directional magnetic flux density.

The Biot–Savart law is modified by replacing the current density with the average current density. Assuming that the average volumetric current density of region A1 in the *x*- and *y*-directions is $J_{aax1}$ and $J_{aay1}$, respectively, the resulting magnetic flux density produced by A1 is:

(14) $$B_{axz1}=\frac{\mu_{0}J_{aax1}}{4\pi }\int_{-l_{R2}}^{0}\int_{f_{1}\left(x\right)}^{s/2}\int_{-h_{a}/2}^{h_{a}/2}\frac{y_{0}-y}{{\left[{\left(x_{0}-x\right)}^{2}+{\left(y_{0}-y\right)}^{2}+{\left(z_{0}-z\right)}^{2}\right]}^{3/2}}dxdydz$$

The expression for the *z*-directional magnetic flux density $B_{ayz1}$ generated at the location point by the current flowing in the *y*-direction is:

(15) $$B_{ayz1}=-\frac{\mu_{0}J_{aay1}}{4\pi }\int_{-l_{R2}}^{0}\int_{f_{1}\left(x\right)}^{s/2}\int_{-h_{a}/2}^{h_{a}/2}\frac{x-x_{0}}{{\left[{\left(x_{0}-x\right)}^{2}+{\left(y_{0}-y\right)}^{2}+{\left(z_{0}-z\right)}^{2}\right]}^{3/2}}dxdydz$$

The total *z*-direction magnetic flux density $B_{az1}$ generated by region A1 is

(16) $$B_{az1}=B_{axz1}+B_{ayz1}$$

Considering the magnetic flux density generated by the rail regions R1, R2, and R3 at location point *P*—where the volume current density in the *x*- and *y*-directions of region R1 are $J_{rx1}$ and $J_{ry1}$, respectively—the value range of each region is shown in Table 2. Similarly, the magnetic flux densities generated by the currents in other directions in each region can be obtained. Due to the velocity skin effect on the current during the launch process, the current direction and density are altered. Therefore, using the average density method can result in significant errors.

### 3.2. ‘Calculated Rail’ Method

**Figure 8:**
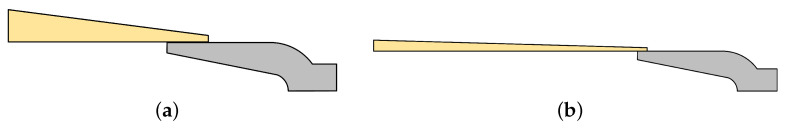
Equivalent model using computational method: (**a**) 1 ms and (**b**) 8.1 ms.

To reduce this type of error, a modeling method is proposed for the shape and contact length of the current distribution area of the rail that can be self-adaptively adjusted. Specifically, based on the distribution feature of the current in the A&R, the actual motion state is simulated by setting the shape of the current distribution area of the rail and the contact length between the A&R. Considering the characteristic of continuous current diffusion in the armature during launch, the armature is modeled according to its actual size. The equivalent model of the A&R shown in Figure 8a,b is established by automatically adjusting the shape and contact length of the ‘calculated rail’. This method is referred to as the adaptive ‘calculated rail’ method.

**Figure 9:**
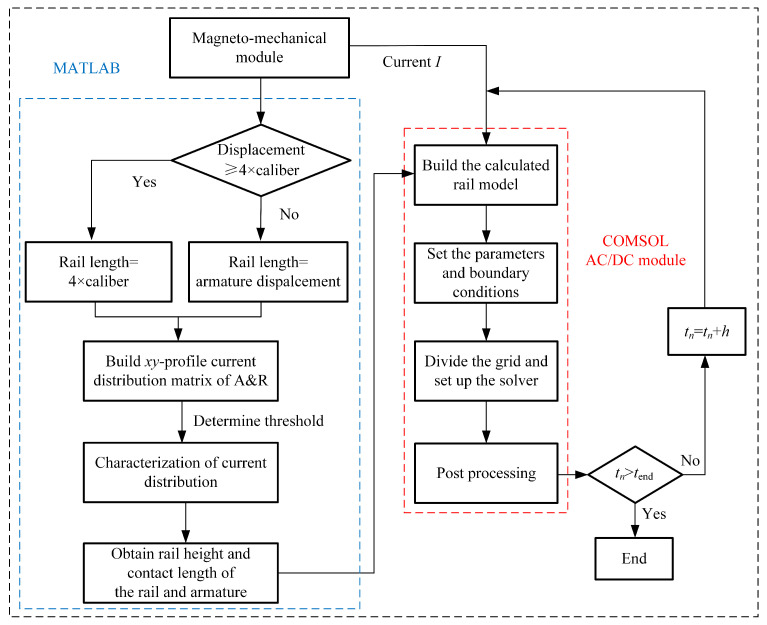
Numerical calculation flow of ‘calculated rail’.

We introduce the adaptive ‘calculated rail’ method into the finite element simulation as shown in Figure 9. The specific calculation method is as follows:

(1) Utilize the circuit–field coupling solver to compute the current distribution within the rail at each time step.
(2) Determine if the displacement of the armature exceeds four times the diameter of the caliber. If it does, set the length of the rail to four times the diameter. Otherwise, set the length of the rail based on the actual armature displacement.
(3) Establish a current distribution map for the rail–armature xy-section based on the actual armature displacement. Export the current distribution map data using a regular grid with a unit area of 0.04 ${\mathrm{mm}}^{2}$ and construct a rail current distribution matrix.
(4) Employ a threshold equal to the average value of the current streamline distribution and apply a binarization technique from image processing to filter the elements in the current distribution matrix. Retain the coordinates with streamline distribution values greater than the mean and eliminate those below the mean.
(5) Construct rail models under the condition of average current based on the results of the binarization filtering.
(6) Utilize the preceding and subsequent heights of the rail as well as the contact length between the rail and armature as input parameters for the COMSOL AC/DC module to establish the ‘calculated rail’ model.
(7) Configure material parameters and boundary conditions with the input current *I* provided by the circuit–field coupling solver.
(8) Perform grid partitioning and solver configuration.
(9) Post-process the solution data to complete one computational cycle.
(10) Execute the computation process for the next time step.

## 4. Results and Discussion

### 4.1. Simulation of Magnetic Field Distribution

**Figure 10:**
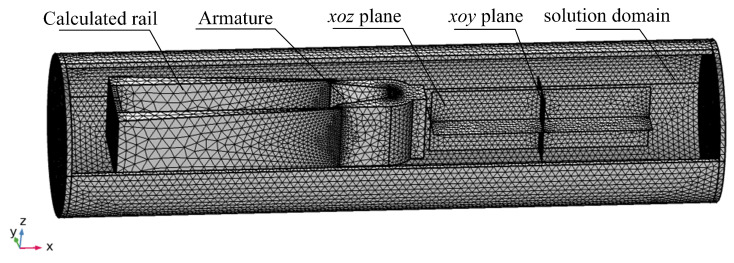
Equivalence model at 8.1 ms.

The rail–armature model at 8.1 ms is shown in Figure 10. The xoy- and xoz-planes have dimensions of 100 mm × 500 mm and 124 mm × 500 mm, respectively. The material parameters of the A&R are consistent with Table 3, while the input current is shown in Figure 4.

**Figure 11:**
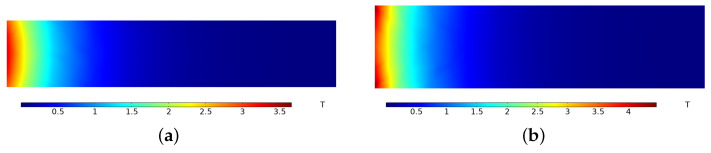
Cloud map of magnetic flux density distribution in front of the armature: (**a**) xoy-plane and (**b**) xoz-plane.

The magnetic flux density distribution at 8.1 ms is shown in Figure 11. Figure 11a shows the magnetic flux density distribution in the xoy-plane at z = 62 mm, where the magnetic flux density diffuses from front to back and the highest magnetic flux density is near the center of the armature, reaching 3.6 T. As the distance from the armature increases, the magnetic flux density decays rapidly, and the magnetic flux density at a distance of 500 mm from the armature is only 0.008 T. Figure 11b shows the magnetic flux density in the xoz-plane at *y* = 50 mm. Unlike the distribution pattern in the xoy-plane, the magnetic flux density is higher at the top and bottom positions, and the peak value reaches 4.2 T.

**Figure 12:**
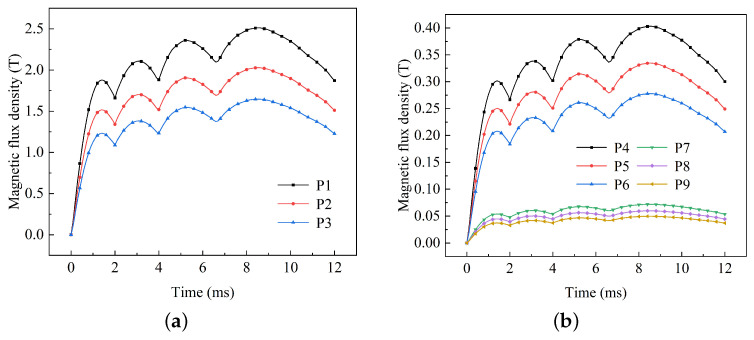
Magnetic flux density at different points in front of the armature: (**a**) P1∼P3 and (**b**) P4∼P9.

Taking into account different placements of the projectile, the points P1∼P9 were established: P1 = 45 mm, P2 = 60 mm, P3 = 75 mm, P4 = 185 mm, P5 = 200 mm, P6 = 215 mm, P7 = 325 mm, P8 = 340 mm, and P9 = 355 mm. These points correspond to the potential locations within the launch load where electronic systems can be placed. The distribution of the magnetic flux density at various points in front of the armature is shown in Figure 12. The magnetic flux density at various points is approximately affected by the variation to input current with time. As time progresses, subsequent pulses exhibit a slightly higher peak magnetic flux density than the first pulse. The maximum magnetic flux density of 2.52 T is achieved at P1 at 9 ms. A peak magnetic flux density of 0.405 T is observed at position P4 in the middle of the projectile, while the peak magnetic flux density at the bottom position, P7, is only 0.06 T.

**Figure 13:**
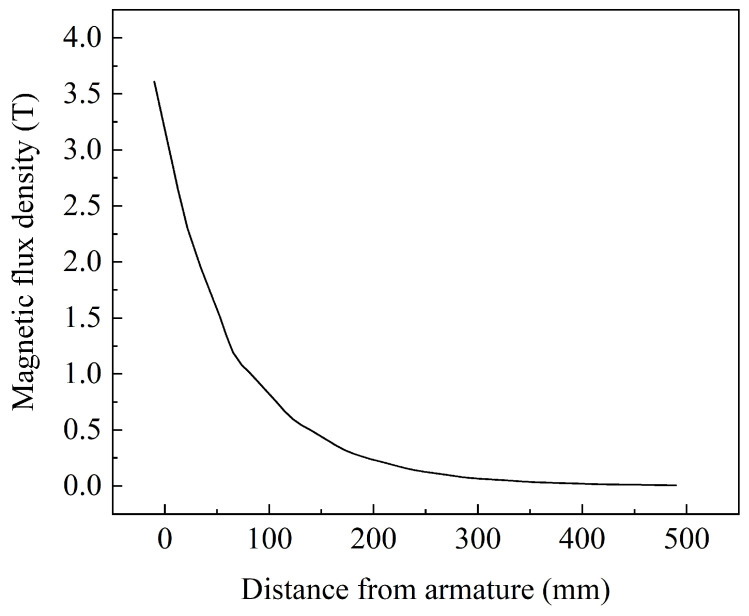
The change in magnetic flux density at different positions at 9 ms.

**Figure 14:**
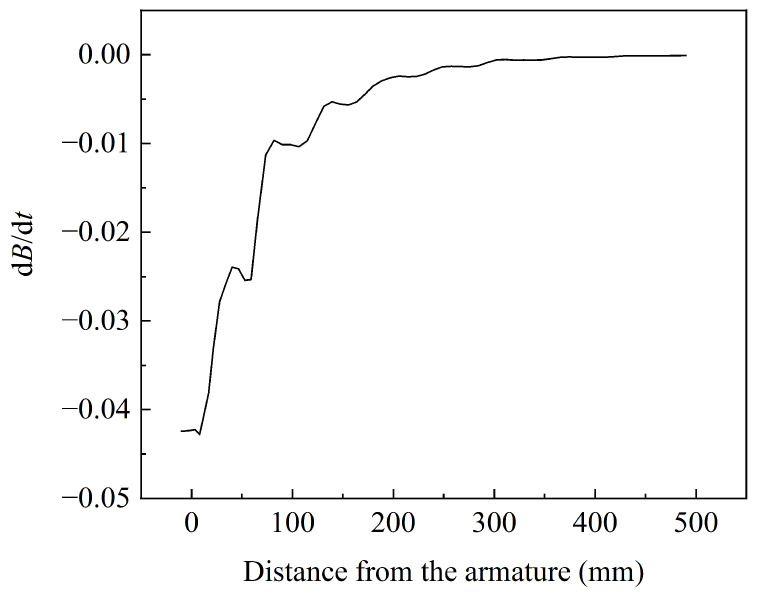
The change rate of magnetic flux density with distance.

At the peak time of 9 ms, the change in the magnetic flux density at different positions is depicted in Figure 13. As the distance between the location point and the armature increases, the magnetic flux density rapidly decreases. The peak magnetic flux density at the top surface of the armature reaches 3.03 T, while at a distance of 490 mm from the armature, the magnetic flux density reduces to only 0.006 T, representing a reduction of over 99%. The change rate of the magnetic flux density with distance is illustrated in Figure 14, where a greater variation in magnetic flux density is observed at different positions in the proximity of the armature.

It can be observed that during electromagnetic launch, although the load is not in the launch circuit of the A&R, the magnetic flux density amplitude is far higher than that of the geomagnetic field (0.03∼0.04 mT) due to the influence of armature and rail magnetic diffusion. The peak magnetic flux density at the bottom of the projectile reaches 2.52 T, while at the middle and head positions it reaches 0.405 T and 0.06 T, respectively. Moreover, the magnetic flux density in the projectile-borne electric system exhibits significant variations. It shows four pulse peaks that are similar to the distribution characteristics of the input current. The initial peak value is relatively small, and the subsequent peak value gradually increases, reaching a maximum at the last pulse peak.

### 4.2. Comparison and Analysis

The existing methods for solving load position differ in two aspects: the input current model and the equivalent model of the A&R. In this section, the solving method used in the previous literature is adopted with the same physical parameters as in the previous section. The calculation errors of these methods are compared, and the reasons for the differences are briefly analyzed.

#### 4.2.1. The Function of Different Input Currents

**Figure 15:**
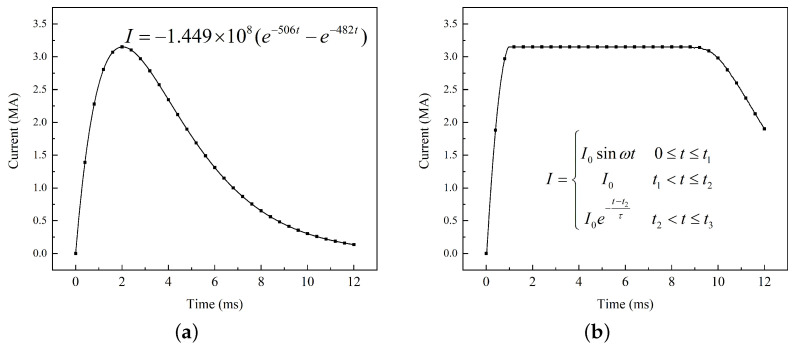
Different current inputs: (**a**) Bi-exponential current and (**b**) three-segment current.

Taking into account the relationship between the magnetic field diffusion and input current, it is important to note that the input current directly affects the simulation results. The commonly used input current is equivalent to a Bi-exponential [28] or a three-segment function [29] based on test results, as illustrated in Figure 15. The total duration is set to 12 ms and the peak current value reaches 3.15 MA.

**Figure 16:**
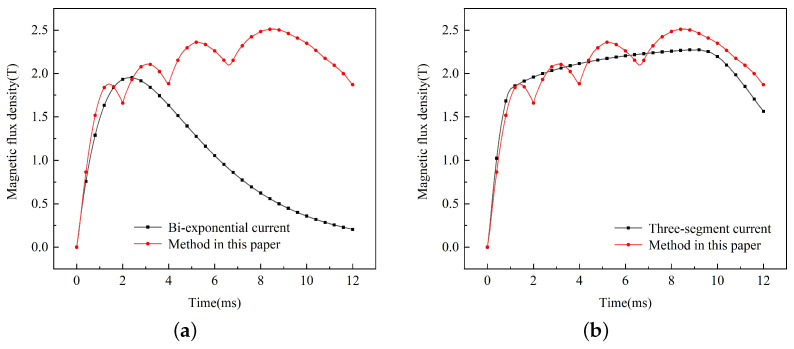
Magnetic flux density calculated by different methods: (**a**) Bi-exponential current and (**b**) three-segment current.

Two types of currents were applied to the model, and the magnetic flux density at the 60 mm position was obtained, as shown in Figure 16. It can be seen that the Bi-exponential current method can reproduce the change frequency in the initial stage, but there is a significant difference from the simulation results of actual multi-pulse currents due to the single-pulse distribution. The simulation results of the three-segment current have a similar characteristic in the initial and final stages, but the ripple effect during the peak stage is not considered, which leads to a large error by replacing it with a constant value.

#### 4.2.2. Different Equivalent Models

**Figure 17:**
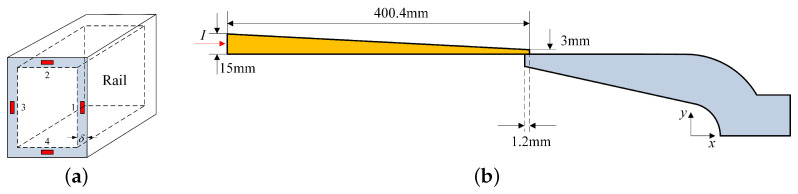
Methods of the previous literature: (**a**) surface current and (**b**) mean rail.

For the magnetic flux density calculation at the load position, this study compares the proposed method with the surface current method [30] and the mean rail method [19] used in the previous literature. The surface current method simplifies the rail model based on the direction of the current flow, as shown in Figure 17a. The mean rail method takes the average of the distribution parameters of the rail current at different times based on the skin depth and the flow direction of the current, as shown in Figure 17b.

**Figure 18:**
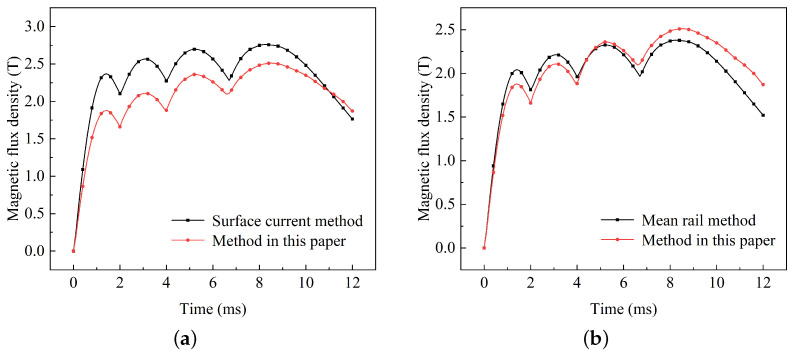
Comparison of simulation methods: (**a**) surface current and (**b**) mean rail.

Utilizing the surface current method, the results obtained at a distance of 60 mm from the electrode are depicted in Figure 18a. The calculated magnetic flux density is higher than that achieved through the method in this paper. The greatest disparity is observed at the first peak, amounting to 18%. This discrepancy primarily arises from the initial low velocity, where the current remains unaffected by the velocity skin effect due to its relatively low speed, resulting in a significantly greater penetration depth compared to the surface. As the velocity increases, the skin depth decreases, and the disparity with the approach detailed in this paper gradually diminishes. Consequently, the surface current method fails to provide an accurate representation and is unsuitable for simulating dynamic electromagnetic emission processes.

The results obtained through the mean rail method are illustrated in Figure 18b. During the initial phase, the magnetic flux density exceeds that of the method in this paper, with the error being 7%. The primary reason for this deviation is the relatively thick skin depth of the current in the initial phase, resulting in the mean rail depth being greater than the actual depth. Conversely, during the later phases, the magnetic flux density falls below that predicted by the methodology in this paper, with an error of 5%. This is primarily attributed to the increased velocity, leading to the mean rail depth being lower than the actual depth. Therefore, the mean rail method employs an average of the current distribution shape rather than capturing the actual dynamic changes, making it an imprecise representation. Both of these methods are unable to precisely represent the magnetic field distribution at the location of loads during dynamic processes.

**Figure 19:**
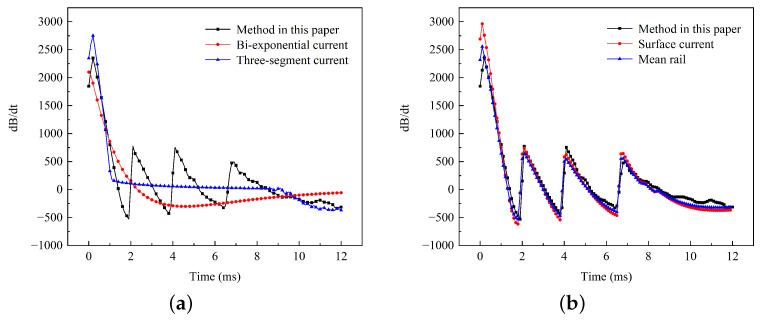
Comparison of the magnetic flux change rate: (**a**) different input currents and (**b**) different equivalent models.

Considering that the pulse magnetic field environment can easily influence internal electronic components of the load in the form of coupled induced voltage, a comparison of the magnetic flux density rate of change obtained by the five methods mentioned above (including the method described in this paper) is shown in the graph. As shown in Figure 19a, the method presented in this paper exhibits three distinct peaks and valleys In the 1∼9 ms range, with peak values reaching 752 and valley values reaching −513. On the other hand, the other two methods show relatively smooth curves that do not accurately represent the pulse characteristics. This alternating induced voltage is more likely to impact electronic components.

The comparison results of the other two methods are shown in Figure 19b. Although they exhibit similar trends to our method in the 1∼9 ms range, there are notable differences, with a maximum error of up to 27%. Thus, those methods cannot accurately calculate the voltage variation curve, resulting in only marginal improvement in the protection of electronic circuits.

Furthermore, a comparative analysis is conducted to assess memory size and estimation time among these various methods during the simulation. Previous methods involved building stationary armature models using COMSOL software and then applying dynamic current loading. The main differences lie in the input currents and equivalent models, with similar computational memory and speed. Consequently, the mean rail method and the method in this paper are selected for comparison, as shown in Table 4.

COMSOL Multiphysics 5.6 is used by the mean rail method with the default finer mesh and a time step of 0.025 ms. An Intel i7-12700 CPU and 32 GB of RAM are used in the simulation. The memory usage during the stationary phase is 10 GB, and during the simulation, it is 18.6 GB, taking approximately 2 h and 3 min to complete. The method in this paper employed COMSOL 5.6 and MATLAB R2020a, utilizing the co-simulation software COMSOL Multiphysics 5.6 with MATLAB for simulation. The mesh partitioning and time step are consistent with the comparative method. Memory usage during simulation was 16.9 GB, and the calculation time was approximately 1 h and 34 min. Therefore, in both of these aspects, the method in this paper also holds a competitive advantage.

### 4.3. In-Bore Dynamic Measurement

In order to verify the accuracy of the simulation model, the magnetic flux density at the load position during electromagnetic launch was obtained using a projectile-borne-storage method. The testing system integrates sensors, signal processing, and storage circuits into a module, which is encapsulated and loaded into the launch load. During the launching process, the load moves with the armature, and the internal storage device continuously tests the characteristics of the magnetic field changes inside the bore and stores the data in memory. After the process is completed, the stored data are read from the testing system using a host computer to complete the projectile-borne-storage process.

**Figure 20:**
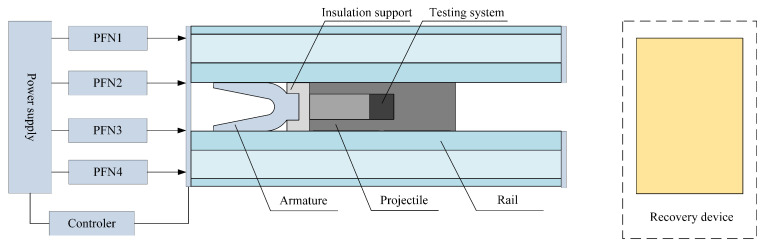
The magnetic field environment testing plan for the electromagnetic launch.

**Figure 21:**
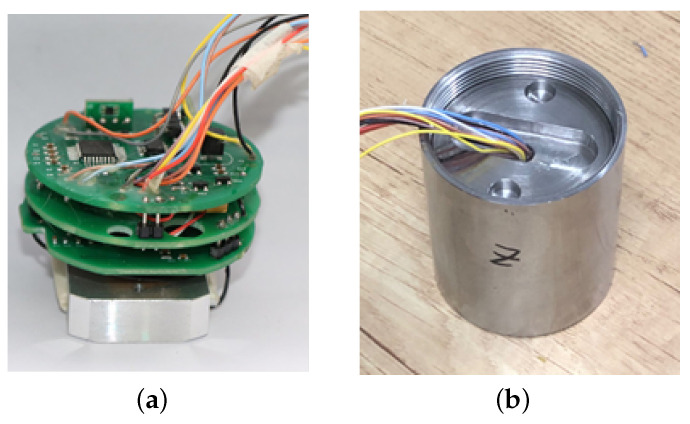
Magnetic field test system: (**a**) assembly circuit and (**b**) after encapsulation.

The electromagnetic railgun consists of four PFNs that supply power to the launching system along with a multi-layer sand recovery device, as shown in Figure 20. The testing system is placed inside the launch load, and the magnetic sensor is positioned in front of the armature. Three linear Hall sensors are placed orthogonally inside the testing system, with a measuring range of 0∼3 T. The external part is encapsulated using polyurethane with a density of 0.6 g/${\mathrm{cm}}^{3}$, as shown in Figure 21.

**Figure 22:**
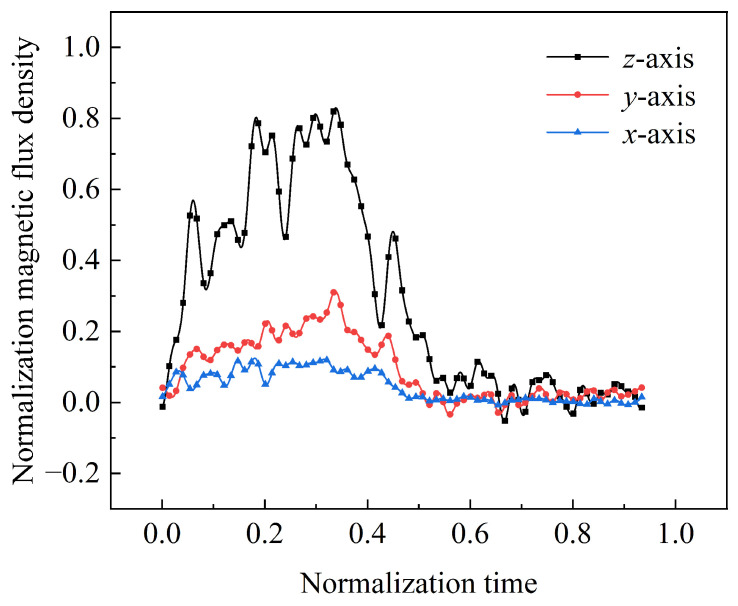
Triaxial magnetic flux density obtained from the experiment.

The three-axis magnetic flux density distribution obtained from experimental testing is presented in a normalized form, as shown in Figure 22. Differently colored lines in the figure correspond to the three-axis magnetic field signals, where time 0 represents the start of the launching stage and time 0.40 represents exiting the muzzle. The black line represents the magnetic flux density along the main axis, with a peak value of 0.84 at time 0.34, exhibiting four distinct pulse peaks. The red and blue lines represent the magnetic field distribution along the other two axes, with peak values of 0.31 and 0.13, respectively, at times 0.34 and 0.32. Each axis has a magnetic flux density distribution with similar characteristics.

### 4.4. Comparison and Discussion

**Figure 23:**
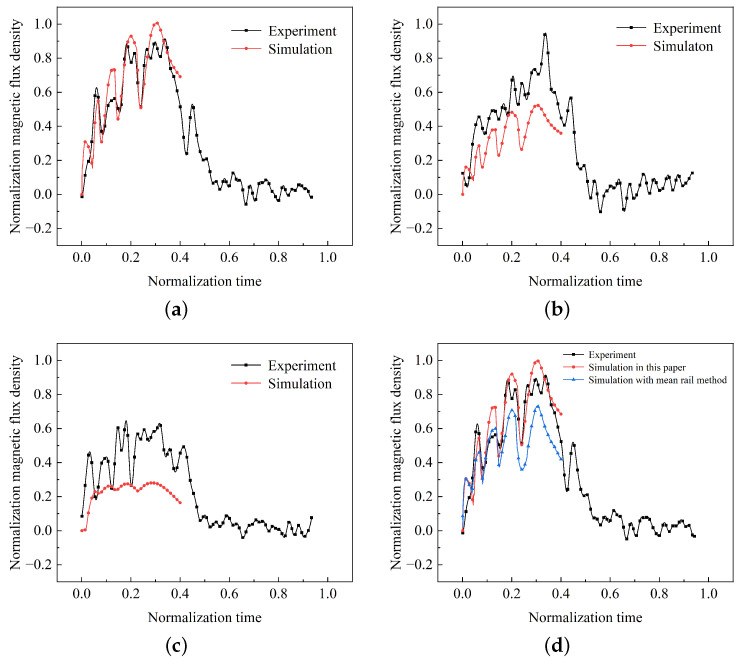
Triaxial simulation values: (**a**) *z*-axis, (**b**) *y*-axis, and (**c**) *x*-axis; (**d**) comparison of *z*-axis.

The simulation model in this paper was established under the same conditions as the experiment, and the results of the three-axis magnetic field simulation were compared with those of the experiment, as shown in Figure 23. To effectively protect the internal circuit components of the test system, the shell is made of low-carbon steel 1008. For simulation purposes, a shell model of the projectile-borne testing system is created, taking into account the shielding effect of the shell material on the pulsed magnetic field.

The comparison of magnetic fields along the main axis is shown in Figure 23a. The simulated and tested results regarding the time, relative magnitude, and trend of multiple peak and valley values calculated based on the pulsed current are generally consistent. Additionally, the peak times of the two curves are close. However, the overall peak value of the simulated magnetic field is greater than the experimental value, resulting in an average error of 7.8%.

The comparison of magnetic fields along the other two axes is shown in Figure 23b,c as well. Similarly, the variations in peak and valley values correspond closely to the test results, but the simulated results are smaller. This discrepancy may be due to the fact that the simulation process did not consider the current density component in the *z*-axis direction, resulting in some errors when calculating the magnetic fields along the remaining two axes. Another possible reason is that the experimental position is far from the current source position of the armature, and during the dynamic motion process, the magnetic field direction is no longer perpendicular to the projectile axis, resulting in a certain angle and an increase in the magnetic field components along the other two axes.

Due to the similarity in the calculated results between the mean rail method and the approach described in this paper, a comparison with the measured data is shown in Figure 23d. Both methods yield magnetic field peak times that are relatively close to the measured results. However, there are discrepancies in the peak and valley values obtained by the mean rail method compared to the measured results, and these differences tend to increase with time.

## 5. Conclusions

In this paper, a method for calculating the magnetic flux density of the load position during electromagnetic launch is presented. The magneto–mechanical coupling model is solved, and the transient current distribution of the A&R is extracted to construct an adaptive and variable ‘calculated rail’ model. The measured data are obtained using the method of a projectile-borne storage system and are compared with the simulation results. The main content and conclusions are as follows:

(1) The magnetic flux density at the load position decreases as the distance from the armature increases, and the initial rate of decrease is rapid. The flux density curve exhibits pulses corresponding to the number of input currents, with the maximum value occurring at the peak of the final pulse.

(2) Compared to the Bi-exponential current and three-segment current methods, the force–magnetic environment coupled with the equivalent circuit calculation method proposed in this paper is more accurate. Additionally, the calculation rail method proposed in this paper greatly reduces the error compared to the surface current model and mean rail model, as the main axis error is only 7.8%;

(3) The measured results show magnetic field distributions in the other two axes that differ from the simulation results. The main reason is the neglect of the *z*-axis current density distribution and its impact in the model, which should be considered in future research.

## Figures and Tables

**Table 1:** Parameters of rail and armature.

Number	PFU Module	Trigger Time (ms)
1	30	0
2	12	2
3	12	4
4	12	7

**Table 2:** The range of values for each integration area.

Direction	R1	R2	R3
*x*	(−($l_{R1}$ + $l_{R2}$), −$l_{R1}$)	(−$l_{R1}$, 0)	(0, $l_{R3}$)
*y*	($s/2,s_{R}$)	($s/2,s_{R}$)	($s/2,s_{R}$)
*z*	(−$h_{r}/2$, $h_{r}/2$)	(−$h_{r}/2$, $h_{r}/2$)	(−$h_{r}/2$, $h_{r}/2$)

**Table 3:** Parameters of the A&R.

Parameter	Rail	Armature
Density $\left(\mathrm{kg}/m^{3}\right)$	8900	2800
Electric conductivity (S/m)	$4.5\times 10^{7}$	$3.5\times 10^{7}$
Relative permeability	1	1

**Table 4:** Comparison of the memory size and estimation time.

Method	Memory Size	Estimation Time
Mean rail method	18.6/32 GB	2 h 3 min
Method in this paper	16.9/32 GB	1 h 34 min

## Data Availability

The data are not publicly available due to the ongoing research.

## Abbreviations

The following abbreviations are used in this manuscript:

A&R	Armature and rail
AC/DC	Alternating current/direct current
Bi	Bilinear interpolation
PFN	Pulse-forming network
PFUs	Pulse-forming units
